# Assessment of Bone Mineral Density, Total Body Composition and Joint Integrity in Long COVID: A 12-Month Longitudinal Feasibility Study

**DOI:** 10.3390/jcm14238558

**Published:** 2025-12-02

**Authors:** Fahad Alghamdi, Abasiama Dick Obotiba, Robert Meertens, Omar Alshalawi, Kinan Mokbel, William David Strain, Karen M. Knapp

**Affiliations:** 1 Department of Radiologic Technology, College of Applied Medical Sciences, Qassim University, Buraydah 52571, Saudi Arabia; 2 College of Medicine and Health, University of Exeter, Exeter EX1 2LU, UK; a.obotiba@exeter.ac.uk (A.D.O.); r.m.meertens@exeter.ac.uk (R.M.); oa369@exeter.ac.uk (O.A.); k.a.mokbel@exeter.ac.uk (K.M.); k.m.knapp@exeter.ac.uk (K.M.K.); 3 Clinical and Biomedical Sciences, Faculty of Health and Life Sciences, University of Exeter, Exeter EX1 2LU, UK; d.strain@exeter.ac.uk

**Keywords:** long COVID, bone mineral density, total body composition, dual-energy X-ray absorptiometry, bone turnover markers, joint health, ultrasound

## Abstract

**Background/Objectives**: A subset of individuals develops persistent symptoms following SARS-CoV-2 infection, including musculoskeletal (MSK) manifestations, a condition known as long COVID (LC). Emerging hypotheses suggest that chronic low-grade inflammation in LC may impair bone metabolism and compromise joint health. However, empirical evidence is limited, and the impact of LC on MSK health, particularly bone and joint integrity, is poorly understood. To determine the influence of LC on MSK function, including bone health, body composition, and joint integrity. **Methods**: A 12-month longitudinal prospective cohort feasibility study was conducted involving 45 adults with LC and 40 well-recovered (WR) post-COVID-19 controls. Baseline and follow-up assessments included dual-energy X-ray absorptiometry (DXA) for bone mineral density (BMD) and total body composition (TBC), alongside ultrasound of the hand and knee joints to evaluate intra-articular changes. **Results**: The LC group had more fat in the gynoid, android, and leg regions at each assessment point compared to the controls (*p* < 0.01). LC showed a significantly lower knee synovial hypertrophy at the baseline, 13.3% compared to WR 45% (*p* = 0.001), and a marginal improvement in hand synovial hypertrophy, over 12 months, from a median of 2 (IQR 1;5) to 1 (IQR 0;3) (*p* = 0.012), as observed via MSK ultrasound. No notable differences were found between groups regarding BMD, either in the LC group compared to the control group or overtime. **Conclusions**: This cohort study of LC adults and controls found no evidence of rapid bone loss; however, adiposity and joint symptoms suggest the need for ongoing monitoring. Future research should focus on MSK markers, muscle function, advanced imaging, and improving MSK health.

## 1. Introduction

In 2019, coronavirus disease 2019 (COVID-19) evolved into a global pandemic caused by the severe acute respiratory syndrome coronavirus 2 (SARS-CoV-2), resulting in widespread morbidity and mortality [1]. Although the majority of infected individuals recover from the acute phase within weeks or a month, an estimated 10% develop prolonged sequelae and complications in their physical health and well-being that last beyond 12 weeks, a condition collectively termed “long COVID” [2,3,4]. The World Health Organisation (WHO) and the National Institute for Health and Care Excellence (NICE) define long COVID (LC) as a multisystem disorder that can involve the respiratory, cardiovascular, neurological and musculoskeletal (MSK) systems [5,6].

Emerging evidence suggests that LC may have significant implications for MSK health, affecting bones, joints and body composition. Individuals experience pain in their spine, reporting pain that is in various locations along the back, or with lower back pain or neck pain, which significantly impacts their quality of life [7,8,9,10,11,12,13,14]. Previous research has reported post-viral MSK deterioration [15,16]. SARS-CoV-2 infection can dysregulate bone metabolism, impair osteoblast function and increase osteoclast-mediated bone degradation [17,18]. These mechanisms raise concerns about increased risk for osteopenia, osteoporosis and fragility fractures in LC patients. Moreover, changes in body composition have been observed following SARS-CoV-2 infection, with evidence suggesting a shift toward higher adiposity and decreased lean muscle mass [16,19].

Many individuals with LC report persistent myalgia, arthralgia and generalised fatigue, resembling post-viral syndromes [20,21,22]. Inflammatory mediators have been implicated in prolonged systemic inflammation, potentially contributing to bone resorption, reduced bone mineral density (BMD) and joint pathology [23]. Given that altered body composition is associated with both osteoporosis and sarcopenia, understanding these changes in LC patients is crucial for early intervention.

While previous studies have explored the respiratory, neuro, and cardiovascular sequelae of LC [24,25,26,27], research on its effects on bone and joint health remains limited. Findings from a recent systematic review indicate that COVID-19 may influence bone health, leading to increased bone regulatory markers, increased bone resorption, reduced bone formation, and decreased BMD. Furthermore, robust imaging studies examining MSK function in LC are not available [28]. This study aims to investigate the impact of LC on MSK function, encompassing bone health, total body composition, and joint integrity.

## 2. Materials and Methods

### 2.1. Study Design and Ethics Approval

The details of the study population with inclusion/exclusion criteria have been described previously in our earlier publication [29]. The Yorkshire and the Humber approved this prospective, longitudinal feasibility study–Bradford Leeds Research Ethics Committee (Reference: 23/YH/0031) on 12 April 2023. Written informed consent was obtained from all participants.

### 2.2. Study Population

Participants were recruited through LC clinics, ongoing LC-related studies, and public advertisements distributed via leaflets, posters, and social media. A total of 85 individuals were enrolled. They were allocated into two groups: the LC group and the Well-recovered group (WR). The inclusion criteria were adults aged 18 years or older of any gender and ethnicity. WR participants had a history of SARS-CoV-2 infection confirmed via RT-PCR or antigen testing. LC participants met the WHO and NICE definitions of long COVID or had a confirmed diagnosis of LC.

The exclusion criteria included:

- Participants who had hospitalisation due to COVID-19 requiring intubation, ICU admission, or ventilatory support (to exclude post-intensive care syndrome);
- Individuals with pre-existing osteoporosis or metabolic bone diseases (e.g., primary hyperparathyroidism, osteogenesis imperfecta);
- Those undergoing long-term corticosteroid therapy (≥5 mg prednisolone daily) or taking bisphosphonates, denosumab, or teriparatide;
- Pregnant or breastfeeding women, due to the use of ionising radiation in DXA scans;
- Participants with recent fractures (<12 months) or conditions affecting joint health, such as rheumatoid arthritis (RA) or systemic lupus erythematosus (SLE).

### 2.3. Data Collection and Assessments

#### 2.3.1. DXA Assessment

BMD and TBC were measured using dual-energy X-ray absorptiometry (DXA) (Lunar Prodigy Advance 2005, GE, Milwaukee, WI, USA) with Encore 11.40.004 software, following the ISCD guidelines for scan acquisition [30]. Quality control (QC) scans were performed using the manufacturer’s calibration phantom before participant assessment to ensure measurement accuracy and minimise precision error [31]. Scans were conducted in accordance with standard clinical positioning protocols [32], including the lumbar spine (L1–L4), femoral neck, and bilateral total hip. Vertebrae exhibiting structural abnormalities or a T-score deviation >1.0 compared to adjacent vertebrae were excluded from the analysis [33]. Osteopenia and osteoporosis were classified using WHO T-score thresholds.

Fracture Risk Assessment (FRAX^®^) and QFracture^®^ tools were used to estimate the 10-year probability of fractures [34,35]. The measurements were based on the WHO osteoporosis T-score classification and the NICE guidelines for fragility fracture risk assessment, observing healing responses, and having a reliable reference range [36,37,38]. TBC included lean mass, fat mass, and fat%. A single operator (FA) conducted all DXA scans, and Prof. (KK) undertook reporting.

#### 2.3.2. Ultrasound Assessment

MSK ultrasound scans of the hand and knee joints were performed using a high-resolution system (Canon Medical System Co., Apilo α I900) equipped with an 18L7 MHz multi-frequency linear array transducer. Grey-scale images were acquired at a scan frequency of 14 MHz, with frame rates of 32 fps for the hand and 26 fps for the knee, and a gain setting of 85. PD signal was assessed using a colour frequency of 7.2 MHz at 16 fps, a pulse repetition rate of 10.7 kHz, and a colour gain of 43. The focal zone was aligned with the region of interest (ROI) for all scans, and the lower end of the Doppler box was positioned as close to the ROI as possible when assessing the PD signal.

For hand ultrasound, the 2nd–5th metacarpophalangeal joints (MCPJs) and proximal interphalangeal joints (PIPJs) (8 joints per participant) were scanned using a modified protocol based on the European Alliance of Associations for Rheumatology (EULAR) guidelines for standardised MSK ultrasound in Rheumatology [39]. A minimum of three images per joint were captured for scoring: longitudinal midline, cross-sectional, and one Power Doppler (PD) signal view. All ultrasound scans were performed and images evaluated at least one month post study visit by one of the authors (FA) under the guidance of ADO, an experienced MSK sonographer [40].

Inter-rater reliability was assessed using greyscale images from 10 randomly selected participants. Two trained assessors (FA and OA), both trained by the same person (ADO), independently scored the images consecutively on the same day. Semi-quantitative grading reliability was assessed using weighted kappa (kw) statistics, while binary outcomes were evaluated using unweighted kappa statistics.

Reliability analysis [Supplementary Table S1] demonstrated substantial to almost perfect agreement across ultrasound features: synovial hypertrophy, synovial effusion, PD signal based on Landis and Koch classification (k: 0 = poor; 0.01–0.2 = slight; 0.21–0.4 = fair; 0.41–0.6 = moderate; 0.61–0.8 = substantial; 0.81–1.0 = almost perfect) [41].

Joint abnormalities were graded based on the Outcome Measures in Rheumatology (OMERACT-7) criteria and definitions [42,43]:

- Synovial hypertrophy: Abnormal hypoechoic intra-articular tissue that is non-displaceable and poorly compressible, and which may exhibit a Doppler signal.
- Synovial effusion: Abnormal hypoechoic or anechoic intraarticular material that is displaceable and compressible but does not exhibit a Doppler signal.
- PD signal intensity: Area of colour signal within the joint capsule in the absence of background noise. Only when there is hypoechoic synovial hypertrophy.

Each feature was graded on a scale from 0 (none) to 3 (severe) [44]. Then the sum scores for each feature were calculated by summing the individual scores from the scanned joints. For each participant, the scores ranged from 0 to 24, where a score of 0 indicated a complete absence of the feature across all joints, and a score of 24 reflected grade 3 severity in all assessed joints for that feature. All acquired images were viewed for scoring using MicroDicom DICOM Viewer 2024.2 (64-bit), which is unlicensed for commercial use and utilises a window-level preset (WL77/WW160).

For the knee joint, the participant lay supine, and the suprapatellar recess was scanned with the knee in flexion of approximately 30° (on either the dominant or most painful side). Ultrasound-detected synovial changes were defined using the OMERACT-7 definitions [42]. Synovial hypertrophy and effusion were measured at their maximum diameter in millimetres using the longitudinal axis. Synovial effusion and hypertrophy were assessed along a longitudinal axis, with an abnormality threshold set at ≥4 mm as recommended by EULAR [45]. PD assessments were conducted in a longitudinal plane, and these pathological phenotypes were scored binary based on whether it was present or absent. The examination protocol adhered to the MSK Ultrasound Technical Guidelines for the knee developed by the European Society of MSK Radiology (ESSR) [46].

### 2.4. Statistical Analysis

Data were analysed using Stata v18.0 (StataCorp, College Station, TX, USA). Given the exploratory nature and small sample size, the analysis focused on group comparisons and within-group changes over 12 months. Independent *t*-tests (or Mann-Whitney U tests for non-parametric data) were used for between-group comparisons, and paired *t*-tests (or Wilcoxon signed-rank tests) for within-group changes. Continuous data were expressed as mean ± standard deviation (SD) or median with interquartile range (IQR), depending on the distribution.

DXA-derived BMD parameters are assumed to follow a normal distribution [47,48]. For this purpose, a *t*-test was conducted to compare the groups, using paired *t*-tests for only the completed records within each group (LC or WR) to assess changes from baseline to follow-up. For the DXA TBC, normality was checked. For normally distributed variables, the difference between the means of two groups was compared using a *t*-test. For non-normally distributed variables, the Mann-Whitney U test was used to compare the difference in medians. A Wilcoxon signed-rank test by group (LC or WR) was used to compare the change within the groups after follow-up, based on only the completed records.

Sum scores of individual ultrasound features (hypertrophy, effusion, and Power Doppler) were treated as continuous data for statistical analysis. Mann-Whitney U-tests were used for non-parametric comparisons. knee ultrasound data were analysed using Chi-square (χ^2^) tests for binary outcomes. Within-group changes were assessed using Wilcoxon rank sum tests for hand joints and McNemar tests for the Knee, reporting the number of participants with discordant changes [positive-to-negative (improved) and negative-to-positive (worsened)].

A significance threshold of *p* < 0.01 was applied to reduce Type I error risk due to multiple comparisons. No formal correction for multiple testing was performed, as analyses were exploratory. Effect sizes and 95% confidence intervals were reported where applicable to indicate the magnitude and precision of observed differences.

#### Power Calculation

The primary objective of this feasibility study is to examine DXA-derived BMD (lumbar spine and total hip) at baseline between participants with LC and WR controls, and to explore changes over 12 months and their association with musculoskeletal symptoms. Using G*Power version 3.1.9.7 (with a two-tailed independent-samples *t*-test, effect size d = 0.6, α = 0.05, power = 0.80), we estimated a minimum sample size of 90 participants (45 per group).

The International Society for Clinical Densitometry (ISCD) guidelines state that a considerable 30 degrees of freedom scan twice to determine the precision error [49]. However, some studies have suggested that using only 30 subjects is not enough to assess the precision error, and the minimum sample sizes required are between 40 and 45, respectively, to estimate the precision error and reduce misclassification of change in an independent patient population [50,51]. Considering that cognitive behavioural therapy trials in ME/CFS reported a meta-analysis pooled dropout rate of ~22%, therefore, a 26% attrition rate for this feasibility study seems a conservative yet reasonable estimate [52]. The aim is to recruit 57 participants per group (114 in total) at baseline. This will ensure that around 45 participants per group remain at follow-up, providing sufficient precision for feasibility outcomes.

## 3. Results

### 3.1. Demographics and Characteristics

The participants’ characteristics have been summarised in Table 1 and described in detail in our previous paper [29].

### 3.2. Comparison of BMD Between LC and WR Groups

**Figure 1:**
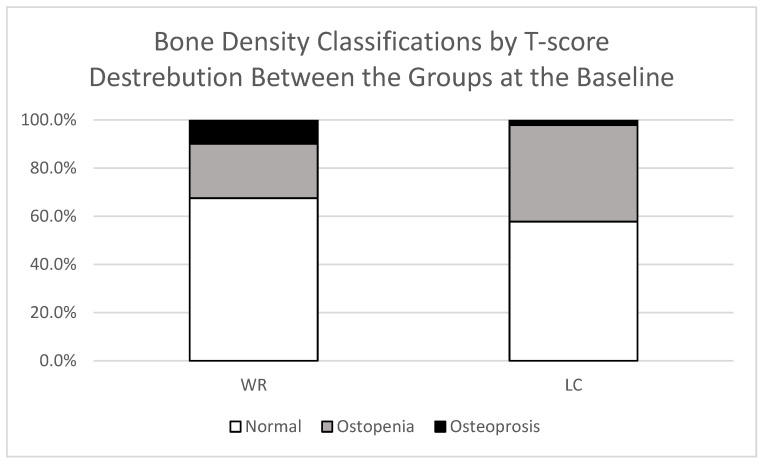
Proportion of participants within each bone mineral density category (normal, osteopenia, and osteoporosis) based on T-scores in the LC (Long COVID) and WR (Well-Recovered) groups.

L1–L4 measurements of 11 LC and 7 WR participants were excluded from the analysis at both timepoints due to scoliosis or degenerative changes caused by vertebrae exhibiting structural abnormalities or a T-score deviation greater than 1.0 compared to neighbouring vertebrae. The baseline BMD measurements, based on T-scores, were slightly higher in the LC group for total body but slightly lower in the lumbar spine and hips compared to the WR group. However, the difference was not significant. Additionally, based on the fracture risk assessment questionnaire, no significant differences have been observed between the groups at baseline, neither in the Major osteoporotic nor hip fracture risk, as shown in Table 2. However, participants with a T-score below −2.5 (osteoporotic range) constituted 2% of the LC group and 10% of the WR group. Additionally, 40% of participants in the LC group and 23% in the WR group were osteopenic (T-score between −1.0 and −2.5), while 58% of the LC group and 68% of the WR group had normal bone density (T-score above −1.0) at the baseline (*p* = 0.103), as shown in Figure 1.

No notable differences were observed between the LC and WR groups at both the start and after 12 months in Total Body, Lumbar Spine (L1–L4), and Hip region BMD [Table 3]. A trend towards lower L1–L4 BMD was observed in LC compared to WR, driven mainly by LC males after sex-stratified subgroup analysis. In L1–L4 BMD, LC males had baseline values of 1.224 ± 0.06 versus 1.298 ± 0.257 g/cm^2^ in WR males (*p* = 0.509), and follow-up values of 1.250 ± 0.119 vs. 1.315 ± 0.3 g/cm^2^ (*p* = 0.651) [Supplementary Table S2]. Additionally, a notable but insignificant decrease in total body BMD over 12 months was seen in the LC male subgroup, decreasing from 1.302 ± 0.075 to 1.285 ± 0.077 g/cm^2^ (*p* = 0.013) [Supplementary Table S3]. Conversely, the LC female subgroup showed slightly higher L1–L4 BMD compared to WR at both time points: baseline (1.200 ± 0.028 vs. 1.146 ± 0.034 g/cm^2^, *p* = 0.248) and at follow-up (1.184 ± 0.157 vs. 1.127 ± 0.144 g/cm^2^, *p* = 0.336), respectively [Supplementary Table S2]. Furthermore, within the WR group, a small but statistically insignificant increase in right total hip BMD (from 1.016 ± 0.171 to 1.023 ± 0.175 g/cm^2^, *p* = 0.025) as shown in Table 4, primarily due to the influence of male subgroups (*p* = 0.0241) [Supplementary Table S3].

### 3.3. Total Body Composition

#### 3.3.1. Gynoid Region

LC participants exhibit a significantly higher percentage of fat tissue and fat mass in the gynoid region than WR participants at both time points. At baseline, the mean gynoid fat percentage was 47.1% in LC vs. 40.2% in WR (*p* < 0.001), and the difference remained significant at follow-up (*p* = 0.001). The mean gynoid fat mass was also higher in the LC compared to the WR groups (6419 ± 2507 g vs. 5171 ± 2040 g; *p* = 0.009), with these differences persisting at follow-up (*p* = 0.008) [Table 3]. Sex analysis revealed that females primarily drove the differences in fat percentage and mass [Supplementary Table S4]. Although the LC group had lower gynoid lean mass compared to the WR group at both baseline (6589 ± 1469 g vs. 7175 ± 1691 g, *p* = 0.088) and follow-up (6653 ± 1609 g vs. 7199 ± 1824 g, *p* = 0.221), the difference was not significant, as shown in Table 3. No significant longitudinal changes in fat or lean mass were observed. However, LC showed a slight, insignificant increase in gynoid fat mass from 6259 ± 2319 g at the baseline to 6355 ± 2221 g at the follow-up (*p* = 0.029) [Table 4], primarily attributed to males (*p* = 0.042), as shown in Supplementary Table S5.

#### 3.3.2. Android Region

In the Android region, LC participants had significantly higher tissue fat percentages than WR at both baseline and follow-up. The mean at baseline was 47.8% in LC compared to 42.6% in WR (*p* = 0.006), and this difference remained significant at follow-up (*p* = 0.006) [Table 3]. These elevated values in LC were primarily due to females (*p* = 0.006), as indicated by sex analyses, as shown in Supplementary Table S4. There were no substantial changes in android fat percentage or fat mass over time within or between groups, as shown in Table 4.

#### 3.3.3. Leg Region and Total Lean Mass

Analysis showed that the LC group had a higher leg fat percentage than the WR participants at both points, primarily due to the female participants [Supplementary Table S4]. At baseline, the LC mean was 43.9%, and the WR mean was 36.3% (*p* = 0.001); at follow-up, the difference persisted (*p* = 0.002) [Table 3]. Leg lean mass was lower in the LC group but only marginally significant at baseline, mean 14,526 ± 3464 g vs. 16,156 ± 3655 g in the WR group (*p* = 0.026), and not at follow-up, the LC group 14,629 ± 3686 g vs. 15,863 ± 3585 g for the WR group (*p* = 0.132) [Table 3]. No significant longitudinal changes within groups, though LC showed a borderline increase in leg fat percentage mean from 43% to 43.7% (*p* = 0.015) [Table 4].

Total lean mass was lower in LC than in WR participants, but the difference was not significant at either baseline or follow-up [Table 3]. No significant changes occurred within groups over time [Table 4].

### 3.4. Intra-Articular Changes

Ultrasound assessment of the hand joints showed no significant differences in synovial hypertrophy scores between LC and WR groups at either baseline or follow-up. However, both groups demonstrated a reduction in synovial hypertrophy scores over 12 months. Despite LC having higher median scores initially, the difference was not statistically significant [Table 3]. Longitudinal changes within-group revealed a marginal reduction in synovial hypertrophy scores in the LC group over 12 months, with the median score decreasing from 2 (IQR 1–5) to 1 (IQR 0–3) (*p* = 0.012), indicating improved intra-articular inflammatory features [Table 4].

Ultrasound assessment of the knee joints revealed that, at baseline, the LC group exhibited significantly less suprapatellar effusion compared to the WR group (13.3% vs. 45.0%, *p* = 0.001) and non-significantly lower synovial hypertrophy (11.1% vs. 32.5%, *p* = 0.016), as shown in Table 3. However, these differences were no longer significant at follow-up (*p* > 0.01). Over time, effusion non-significantly increased in LC from 13.3% to 30.6% (*p* = 0.023), indicating potential changes in the progression of suprapatellar effusion [Table 4].

## 4. Discussion

To address the knowledge gap regarding LC, a longitudinal cohort study was conducted by following adults with LC and WR controls over 12 months. Furthermore, our recent findings suggest that LC is associated with poorer HRQoL, particularly in terms of physical health, and increased joint pain [29]. This also relates to a decline in exercise capacity, which can be partly seen as the natural deconditioning that occurs when someone has been ill. COVID-19 and LC have emerged as significant catalysts for the generalised deterioration of skeletal muscle [53]. This study aimed to investigate whether LC influences MSK function, including bone health, body composition, and joint integrity. This study found significant differences in two strands: between the two groups, LC had increased gynoid, android, and leg fat at baseline compared to WR. Within 12 months, there was an improvement in hand joint synovial hypertrophy in the LC group. Furthermore, no significant differences were observed in BMD. This suggests that factors beyond visible musculoskeletal inflammation, such as deconditioning, metabolic changes, autonomic dysfunction, or central pain mechanisms, may contribute to symptom persistence. Notably, the study provides a 12-month longitudinal perspective, which is limited in the current literature and was identified as a gap by our previous systematic review [28].

### 4.1. Long COVID Associated with Increased Total Body Composition in Both Android and Gynoid Areas

Participants in the LC group exhibited significantly higher fat mass, particularly in the android and gynoid regions, and a trend toward reduced lean mass in the lower limbs compared to the WR controls. These differences persisted after 12 months, likely reflecting reduced physical activity and deconditioning driven by fatigue and pain [54,55]. The combination of increased fat and decreased lean mass may lead to MSK functional decline and elevated metabolic risk, providing a plausible link to poorer HRQoL. COVID-19 pandemic has been crucially linked with body composition, even in young adults [56,57]. Previous studies have demonstrated decreased muscle mass and elevated visceral adipose tissue in both acute and post-COVID-19 phases [16,19,58]. A cross-sectional study revealed that individuals suffering from LC exhibited significantly reduced levels of total and appendicular lean mass [59], which is consistent with the current findings.

Mechanistically, elevated inflammatory markers may accelerate protein breakdown, leading to a reduction in muscle mass, or the MSK tissue becomes an easy target for viruses caused by Angiotensin-converting enzyme two expression, leading to tissue damage [59,60,61]. Yet, we assessed makers such as IL-6, CRP, TNF-α, and IFN-γ, and these showed no significant differences between the LC and control group [29]. Similar patterns are observed in other chronic inflammatory diseases, where excess fat and reduced muscle mass are associated with adverse health outcomes and increased mortality [60,62]. Persistent fatigue and inactivity in LC likely contribute to muscle deconditioning and fat accumulation. Peripheral mechanisms, including both structural and functional muscle damage, have been linked to LC pathophysiology [63,64]. Metabolic disruptions, severe myopathy, and amyloid deposits in muscles had been linked with LC activities [65].

These findings emphasise the importance of targeted rehabilitation strategies to mitigate muscle loss and restore function. Body composition is a modifiable factor in LC. Interventions such as symptom-based physical activity, progressive resistance training, and nutritional support may help reverse adverse changes. However, post-exertional malaise remains a challenge, as increased activity can exacerbate symptoms in some individuals. Further trials are needed to identify safe and effective strategies for preserving MSK integrity in LC populations.

### 4.2. No Association of Bone Mineral Density in Long COVID

No significant differences in BMD were observed between LC and WR participants, with no notable decline in either group over 12 months. These findings suggest that LC did not induce detectable bone loss or disrupt bone remodelling during the study period, consistent with our bone turnover markers finding [29]. Our systematic review highlighted adverse bone effects in acute and post-acute COVID-19, including elevated bone turnover markers and reduced BMD; however, LC-specific data remain scarce [28]. This study is among the first to evaluate bone health in a clearly defined LC cohort. The contrast between the bone loss observed in acute or post-acute COVID-19 cases and the stability seen in the LC participants suggests that skeletal effects may be transient, phenotype-dependent, or driven by acute-phase factors such as inflammation, immobilisation, or corticosteroid use, none of which were applied in this sample.

Mean total body BMD (~1.22 g/cm^2^) remained nearly unchanged after 12 months. Both groups experienced minor total body BMD decline, with LC males showing slightly greater reductions than WR males. Yet, bone health remained stable in the LC sample, which had a prolonged decline in quality of life from the chronic condition and did not receive ongoing high-dose steroids. However, while fracture risk in this specific LC population may be lower than initially thought, continued vigilance is essential over the long term.

An early prediction is that individuals who have contracted COVID-19, as well as the safety measures or lockdown, may face an effect on bone health [66]. Even in young adults with no risk, a study found that the COVID-19 pandemic affected bone tissue, as shown by changes in the radius BMD, indicating a negative impact on bone quality and health [67]. Furthermore, some studies found that non-human models infected with SARS-CoV-2 harm trabecular bone acutely via alterations in bone structure and an increase in osteoclast numbers [68,69,70,71]. While this study did not find a significant difference in BMD between LC and WR individuals, it suggests that the observed trend of lower values in LC participants warrants further investigation in future research.

It has been suggested that corticosteroid use in COVID-19 patients may contribute to these physiological changes. When comparing BMD reduction between non-COVID-19 corticosteroid patients and COVID-19 corticosteroid patients, it was observed that the group with SARS-CoV-2 had lower BMD [72]. In this study population, no participants were on high-dose corticosteroids (over 5 mg of prednisolone or equivalent daily). These findings are reassuring for LC patients without osteoporosis risk factors, highlighting the importance of longer-term follow-up beyond 1 year and targeted studies of higher-risk LC subgroups, such as post-hospitalisation patients, steroid users, and older adults.

### 4.3. Long COVID Linked to Persistent Joint Pain with 12-Month Reduction in Hand Synovial Hypertrophy

Ultrasound findings revealed minor subclinical intra-articular changes in LC participants. Despite this, joint pain scores remained elevated and unresolved after 12 months. Exploratory scans of the dominant or most symptomatic side showed an insignificant but noticeable decrease in hand synovial hypertrophy in the LC group, though no differences were detected compared with the WR group. Synovial effusion or active synovitis, as PD signals remain minimal. Notably, the longitudinal reduction in hypertrophy was consistent with the longitudinal changes in IL-6 observed in our previous results [29]. In osteoarthritis, increased metalloproteinases, synovitis, and cartilage deterioration are linked to inflammatory mediators in the synovium/synovial fluid [73].

At baseline, fewer LC participants had knee effusion than the WR group. However, the LC group showed a non-significant increase in knee effusion at follow-up. Yet, many isolated case reports and very few studies include sonographic assessment. Gasparotto et al.’s case report found that a unilateral articular change in the ankle and knee [74]. Also, Mukarram et al. case series report involving five post-COVID-19 patients who exhibited clinical features resembling rheumatoid arthritis, including grade 2 synovitis in their metacarpophalangeal joints, consistent with rheumatoid arthritis like presentations [75].

This may reflect incidental or transient findings rather than persistent pathology. Viral infections such as hepatitis B or C, Epstein-Barr virus, and HIV are known to affect joints [76,77,78,79], and widespread joint and muscle pain has been reported in acute COVID-19 [80]. Similar patterns were seen in SARS-CoV-1, where persistent joint pain lasted up to four years despite negative MRI findings, suggesting neurogenic pain or undetectable low-grade synovitis [81]. Most cases of SARS-CoV-2 arthritis cases lack viral presence in synovial fluid, nor in deceased COVID-19 patients [82], though one study identified viral nucleic acids in the joints of moderately ill outpatients [83]. However, evidence for direct viral invasion of synovial tissue remains limited [84,85,86].

Two main theories exist: one suggests viral arthritis driven by viremia or cytokine storm [86]; the other suggests reactive arthritis triggered by systemic inflammation [87]. Knee joint effusion with synovitis often causes knee pain in older adults, especially in the suprapatellar compartment [88], and in osteoarthritis, these changes are linked to inflammation of the synovium and synovial fluid, as well as immune activation associated with osteophytes, all of which promote structural progression [73,89]. As LC patients are more prone to a sedentary lifestyle due to the related symptoms [90], this may contribute to muscle weakness and altered joint mechanics. Some participants attempted to cope through increased physical activity; however, the transition from inactivity to activity, combined with underlying muscle deconditioning and a higher BMI, may have predisposed them to knee effusion. Prolonged inactivity in LC can lead to muscle weakness and deconditioning, while a high BMI adds mechanical stress. Together, these factors increase the risk of knee effusion and synovitis, especially when transitioning from inactivity to activity [91,92].

In this LC cohort, objective signs of synovial changes were present despite persistent joint pain, and ultrasound revealed only minor alterations. This suggests that LC MSK pain may not stem from obvious arthritis, but rather from neural mechanisms or subtle inflammation, which guides treatment options such as pain management or rehabilitation. Knee effusion trends may be associated with weight gain, deconditioning of the lower limbs, or resumption of activity after a period of inactivity. These changes are likely driven more by mechanical aspects than by inflammation. Clinically, this suggests that emphasis should be placed on rehabilitation strategies, such as strengthening exercises and load management, rather than on disease-modifying anti-inflammatory treatments for similar LC profiles, unless red flags suggest an alternative approach.

### 4.4. Limitations

This study has several limitations. First, the statistical approach depended on paired and independent tests, overlooking repeated measures or interactions. Moreover, multiple univariate tests were performed without formal correction for multiple comparisons, raising the likelihood of false positives despite applying a stricter *p* < 0.01 threshold. Second, the small sample size, combined with a 22% withdrawal rate, may have reduced the ability to detect subtle effects and introduced attrition bias. Recruitment was voluntary and mainly conducted during winter, which may have introduced bias and increased symptom variability, as well as seasonal effects on activity levels. Third, generalisability is limited because most participants were Caucasian individuals from semi-urban South-West UK, with women overrepresented in the LC group. Lifestyle factors such as diet and exercise were not fully controlled, which could potentially influence body composition outcomes. Fourth, observational design limits the ability to draw causal conclusions. Factors such as unmeasured baseline behaviours, concurrent treatments, and natural recovery processes could have influenced the results, so the observed changes cannot be attributed solely to LC. Finally, the one-year DXA follow-up may not capture meaningful changes in BMD. Some participants starting new treatments might also have affected bone outcomes. The lack of rheumatological assessment, joint autoantibody testing, or genetic analysis further limits the interpretation of potential subclinical inflammatory or degenerative findings. Nonetheless, the data provide a foundation for future research into the underlying mechanisms of LC-related MSK effects.

## 5. Conclusions

This study found that over 12 months, LC was associated with higher TBC in both the android and gynoid regions, and ultrasound showed only slight improvement in hand synovial hypertrophy over time. No significant changes were observed between LC and BMD. These findings suggest that MSK sequelae in LC may develop more subtly and require longer follow-up for detection. While LC does not seem to induce rapid or significant changes in bone density within one year, it has a noticeable effect on fat distribution and joint symptoms. While no definitive evidence was found for accelerated bone loss within the study period, the observed changes in adiposity and joint symptoms underscore the need for ongoing monitoring and care in this population. This study lays the groundwork for future studies incorporating rheumatological biomarkers, muscle function assessments, and advanced imaging to better understand and mitigate the long-term MSK effects of LC.

## Figures and Tables

**Table 1:** BMI: Body mass index (kg/m^2^); yr: years; WR: Well-recovered; LC: long COVID; (*n*): participants number at each timepoint; *p*-values using appropriate tests (Mann–Whitney U or independent *t*-tests) for continued data based on data normality and X^2^ or Fisher’s exact test for categorical data; data are presented as median with interquartile range (IQR), mean ± standard deviation (SD), or *n* (%): number and percentages as appropriate; (₽): *t*-tests; (‡): Mann–Whitney U; (¥): Fisher’s exact test; (X): X^2^ test; * Statistically significant at *p* < 0.01.

Participant Characteristics						
>Variables	Baseline			Follow-up		
	WR (*n* = 40)	>LC (*n* = 45)	> *p*	>WR (*n* = 30)	>LC (*n* = 36)	> *p*
Age (yr) ^(₽)^	51 ± 15.17	52.22 ± 9.94	0.658	52.83 ± 14.85	53.28 ± 10.08	0.885
Sex (Female), *n* (%) ^(X)^	19 (47)	38 (84.45)	<0.001 *	15 (50)	29 (80.56)	0.009 *
BMI (kg/m^2^) ^(‡)^	26.6 (23.8; 30.65)	27.9 (24.7; 33)	0.214	25.55 (23.4; 29.3)	28.8 (23.8; 34.45)	0.087
Ethnicity, *n* (%) ^(¥)^			0.459			1.00
White or not stated	39 (97.5)	41 (91.1)		30 (100)	33 (91.7)	
Indian	0 (0.0)	2 (4.4)		0 (0.0)	1 (2.8)	
Pakistani	0 (0.0)	1 (2.2)		0 (0.0)	1 (2.8)	
Black African	1 (2.5)	0 (0.0)		0 (0.0)	0 (0.0)	
Chinese	0 (0.0)	1 (2.22)		0 (0.0)	1 (2.8)	
Socio-economic, *n* (%) ^(X)^			0.266			0.449
Upper	20 (50)	23 (51.1)		15 (50)	18 (50)	
Upper Middle	13 (32.5)	19 (42.2)		12 (40)	17 (47.2)	
Lower Middle	7 (17.5)	3 (6.7)		3 (10)	1 (1.8)	
Smoking status, *n* (%) ^(¥)^			0.298			0.742
Non-smoker	26 (65)	35 (77.8)		21 (70)	26 (72.2)	
Ex-smoker	8 (20)	8 (17.8)		4 (13.3)	6 (16.7)	
Light smoker (less than 10)	2 (5)	2 (4.44)		2 (6.67)	3 (8.3)	
Moderate smoker (10 to 19)	3 (7.5)	0 (0.0)		1 (3.33)	1 (2.8)	
Heavy smoker (20 or over)	1 (2.5)	0 (0.0)		2 (6.67)	0 (0.0)	
Alcohol status, *n* (%) ^(¥)^			0.526			0.396
Non	15 (37.5)	22 (48.89)		16 (53.33)	21 (58.3)	
<1 unit per day	12 (11.3)	10 (22.2)		7 (23.33)	9 (25)	
1–2 units per day	9 (22.5)	8 (17.8)		4 (13.33)	4 (11.1)	
3–6 units per day	1 (2.5)	4 (8.9)		3 (10)	0 (0.0)	
7–9 units per day	1 (2.5)	0 (0.0)		0 (0.0)	1 (2.8)	
>9 units per day	2 (5)	1 (2.22)		0 (0.0)	1 (2.8)	
Hormonal Replacement Therapy, *n* (%) ^(X)^	3 (7.5)	8 (17.8)	0.159			
Supplementation of Vitamin D, *n* (%) ^(X)^	6 (20)	14 (38.9)	0.080			
Bone Health Medication, *n* (%) ^(X)^	-	-	-	5 (16.7)	1 (2.8)	0.084

**Table 2:** T-score: compares a participant’s bone density to the normal range observed in young healthy adults; WR: Well-recovered; LC: long COVID; (n): participants number at baseline; *p*-values using independent *t*-tests with ± SD for fracture risk Mann–Whitney was used for as not normally distribution with (IQR); Data are presented as mean ± standard deviation (SD); (₽): *t*-tests; (‡): Mann–Whitney U.

DXA T-Score Baseline Results Between the Study Group WR and LC Participants.					
T-score		*n =* (WR/LC)	WR	LC	*p*
Total Body ^(₽)^		(39/45)	0.534 ± 1.379	1.037 ± 1.161	0.073
L1–L4 ^(₽)^		(32/33)	0.244 ± 1.789	0.138 ± 1.184	0.780
Total Hip ^(₽)^	RT	(36/37)	−0.247 ± 1.099	0.015 ± 1.036	0.263
	LT	(39/44)	−0.273 ± 1.174	−0.033 ± 1.043	0.326
Fracture Risk (%)					
Major osteoporotic ^(‡)^		(27/40)	5.7 (3.3; 11.8)	4.85 (2.85; 8.4)	0.165
Hip ^(‡)^			0.5 (0.2; 2.5)	0.45 (0.1; 0.9)	0.221

**Table 3:** BMD: bone mineral density (g/cm^2^); L1–L4: lumbar spine; Rt: Right; Lt: Left; WR: Well-recovered; LC: long COVID; (*n*): participants number completed DXA scan at each timepoints; *p*-values based using appropriate tests (Mann–Whitney U or independent *t*-tests) based on data normality; Data are presented as mean ± standard deviation (SD) as appropriate; for Ultrasound: Mann-Whitney U for hand, and Chi^2^ for the knee; values are presented as medians with interquartile range (IQR) or n (%) respectively; (₽): *t*-tests; (‡): Mann–Whitney U; (X): X^2^ test; * Statistically significant at *p* < 0.01.

Musculoskeletal Imaging Results Comparing WR and LC Participants.										
	Region	Baseline					Follow-Up			
		Side	(*n*)	WR	LC	*p*	(*n*)	WR	LC	*p*
**BMD**	Total body ^(₽)^	-	(39/45)	1.219 ± 0.127	1.223 ± 0.099	0.876	(30/36)	1.215 ± 0.143	1.219 ± 0.104	0.909
	L1–L4 ^(₽)^	-	(32/33)	1.232 ± 0.221	1.204 ± 0.142	0.547	(23/28)	1.233 ± 0.258	1.195 ± 0.151	0.519
	Femoral neck ^(₽)^	Rt	(39/45)	0.967 ± 0.144	0.974 ± 0.134	0.819	(30/36)	0.961 ± 0.144	0.98 ± 0.143	0.605
		Lt	(39/45)	0.969 ± 0.154	0.976 ± 0.152	0.843	(30/36)	0.965 ± 0.157	0.997 ± 0.223	0.519
	Total hip ^(₽)^	Rt	(39/45)	1.026 ± 0.160	1.024 ± 0.138	0.959	(30/36)	1.023 ± 0.176	1.029 ± 0.152	0.885
		Lt	(39/44)	1.023 ± 0.173	1.017 ± 0.135	0.876	(30/34)	1.021 ± 0.188	1.018 ± 0.149	0.938
**TBC**	Gynoid Region Fat (%) ^(‡)^		(39/45)	0.402 ± 0.089	0.471 ± 0.084	<0.001 *	(30/36)	0.399 ± 0.087	0471 ± 0.077	0.001 *
	Gynoid Tissue Fat (%) ^(‡)^			0.411 ± 0.090	0.481 ± 0.085	<0.001 *		0.399 ± 0.0878	0.471 ± 0.077	0.001 *
	Gynoid Fat Mass (g) ^(‡)^			5171 ± 2040	6419 ± 2507	0.009 *		5107 ± 2111	6355 ± 2221	0.008 *
	Gynoid Lean Mass (g) ^(‡)^			7175 ± 1691	6589 ± 1469	0.088		7199 ± 1824	6653 ± 1609	0.221
	Android Region Fat (%) ^(‡),(₽)^			0.421±0.101	0.474 ± 0.112	0.006 *		0.418 ± 0.094	0.483 ± 0.092	0.006 *
	Android Tissue Fat (%) ^(‡),(₽)^			0.426 ± 0.101	0.478 ± 0.112	0.006 *		0.422 ± 0.094	0.487 ± 0.092	0.006 *
	Android Region Fat Mass (g) ^(‡)^			2809 ± 1439	3483 ± 1817	0.067		2731 ± 1481	3524 ± 1761	0.064
	Legs Tissue Fat (%) ^(‡),(₽)^			0.363 ± 0.102	0.439 ± 0.101	0.001 *		0.360 ± 0.106	0.437 ± 0.095	0.002 *
	Legs Lean Mass (g) ^(‡)^			16,156 ± 3655	14,526 ± 3464	0.026		15,863 ± 3585	14,629 ± 3686	0.132
	Total Lean Mass (g) ^(‡)^			48,914 ± 10,060	45,326 ± 10,155	0.054		48,391 ± 10,804	45,763 ± 10,739	0.236
**Intra-Articular**	Hand Synovial Hypertrophy ^(‡)^		(40/45)	2 (1; 4)	3 (1; 5)	0.502	(30/36)	1.5 (0; 3)	1 (0; 3)	0.742
	Hand Synovial Effusion ^(‡)^			0 (0; 1)	0 (0; 1)	0.684		0 (0; 1)	0 (0; 0)	0.212
	Hand Power Doppler ^(‡)^			1 (0; 3)	1 (0; 4)	0.274		1 (0; 2)	0.5 (0; 2)	0.695
	Knee Synovial Hypertrophy ^(X)^			13 (32.5)	5 (11.1)	0.016		7 (23.3)	4 (11.1)	0.185
	Knee Synovial Effusion ^(X)^			18 (45)	6 (13.3)	0.001 *		14 (46.7)	13 (36.1)	0.385
	Knee Power Doppler ^(X)^		(32/42)	0 (0)	0 (0)	1.000	(30/33)	2 (6.67)	2 (6.06)	0.922

**Table 4:** BMD: bone mineral density (g/cm^2^); L1–L4: lumbar spine; Rt: Right; Lt: Left; WR: Well-recovered; LC: long COVID; (*n*): participants number completed assessments at both timepoints; *p*-values based on normality for continuous variables as appropriate paired *t*-test for BMD or Wilcoxon signed-rank; values reported as mean ± standard deviation (SD) or median with interquartile range (IQR) as appropriate; Suprapatella recess Knee Joint Statistical tests used McNemar test, with data presented as numbers with (% change) of discordant pairs relative to participants change positive finding to negative finding and negative finding to positive finding; (P): paired *t*-test; (ω): Wilcoxon signed-rank; (*₼*): McNemar test.

Within the LC and WR Group, Changes in Musculoskeletal Imaging Results.										
**Region**		**Side**	***(n)***	**WR**			***(n)***	**LC**		
				**Baseline**	**Follow-up**	***p***		**Baseline**	**Follow-up**	***p***
**BMD**	Total body ^(P)^	-	30	1.216 ± 0.142	1.215 ± 0.143	0.837	36	1.224 ± 0.106	1.219 ± 0.104	0.068
	L1–L4 ^(P)^	-	23	1.235 ± 0.251	1.233 ± 0.258	0.812	24	1.197 ± 0.144	1.183 ± 0.148	0.173
	Femoral neck ^(P)^	Rt	30	0.958 ± 0.148	0.961 ± 0.144	0.513	36	0.984 ± 0.144	0.98 ± 0.143	0.463
		Lt		0.97 ± 0.16	0.965 ± 0.157	0.310		0.976 ± 0.164	0.997 ± 0.223	0.158
	Total hip ^(P)^	Rt		1.016 ± 0.171	1.023 ± 0.175	0.025		1.028 ± 0.146	1.029 ± 0.152	0.779
		Lt		1.018 ± 0.185	1.021 ± 0.188	0.307	34	1.016 ± 0.146	1.018 ± 0.149	0.494
**TBC**	Gynoid Region Fat (%) ^(ω)^			0.401 ± 0.083	0.399 ± 0.087	0.926	36	0.465 ± 0.085	0.471 ± 0.077	0.055
	Gynoid Tissue Fat (%) ^(ω)^			0.410 ± 0.084	0.399 ± 0.087	0.066		0.475 ± 0.085	0.471 ± 0.077	0.271
	Gynoid Fat Mass (g) ^(ω)^			5187 ± 2110	5107 ± 2111	0.517		6259 ± 2319	6355 ± 2221	0.029
	Gynoid Lean Mass (g) ^(ω)^			7236 ± 1858	7199 ± 1824	0.416		6654 ± 1539	6653 ± 1609	0.851
	Android Region Fat (%) ^(ω)^			0.417 ± 0.097	0.417 ± 0.093	0.228		0.476 ± 0.106	0.482 ± 0.092	0.087
	Android Region Fat Mass (g) ^(ω)^			2792 ± 1539	2731 ± 1481	0.428		3511 ± 1789	3524 ± 1761	0.307
	Android Tissue Fat (%) ^(ω)^			0.421 ± 0.097	0.422 ± 0.094	0.229		0.480 ± 0.107	0.487 ± 0.092	0.102
	Legs Tissue Fat (%) ^(ω)^			0.363 ± 0.099	0.360 ± 0.105	0.585		0.430 ± 0.101	0.437 ± 0.095	0.015
	Legs Lean Mass (g) ^(ω)^			16,134 ± 4006	15,863 ± 3585	0.236		14,754 ± 3652	14,629 ± 3686	0.489
	Total Lean Mass (g) ^(ω)^			48,759 ± 10,980	48,391 ± 10,804	0.089		45,991 ± 10,815	45,763 ± 10,739	0.441
**Intra-Articular**	Hand Synovial Hypertrophy ^(ω)^			2 (1; 4)	1.5 (0; 3)	0.123		2 (1; 5)	1 (0; 3)	0.012
	Hand Synovial Effusion ^(ω)^			0 (0; 1)	0 (0; 1)	0.702		0 (0; 0)	0 (0; 0)	0.139
	Hand Power Doppler ^(ω)^			1.5 (0; 4)	1 (0; 3)	0.481		1 (0; 2)	0.5 (0; 2)	0.228
	Knee Synovial Hypertrophy ^(*₼*)^			4 (66.7)	2 (33.3)	0.687		1 (33.3)	2 (66.7)	1.000
	Knee Synovial Effusion ^(*₼*)^			4 (50)	4 (50)	1.000		2 (15.4)	11 (84.6)	0.023
	Knee Power Doppler ^(*₼*)^		26	0 (0)	2 (100)	0.500	31	0 (0)	2 (100)	0.500

## Data Availability

The original contributions presented in the study are included in the article (and Supplementary Material); further inquiries can be directed to the corresponding author.

## Supplementary Materials

The following supporting information can be downloaded at: https://www.mdpi.com/article/10.3390/jcm14238558/s1, Table S1: Intra-Rater Reliability of Ultrasound Scoring Across Joints; Table S2: Baseline and Follow-up BMD (g/cm^2^) Measured by DXA in LC and WR Participants, Stratified by Sex; Table S3: Sex Stratified Within LC and WR Group BMD Changes (g/cm^2^) from Baseline to Follow-up; Table S4: Baseline and Follow-up Total Body Composition by DXA in LC and WR Participants, Stratified by Sex; Table S5: Sex Stratified Within LC and WR Group Changes Body Composition from Baseline to Follow-up.

## Abbreviations

The following abbreviations are used in this manuscript:

BMD	Bone Mineral Density
DXA	Dual-Energy X-Ray Absorptiometry
LC	Long COVID
MSK	Musculoskeletal
PD	Power Doppler
TBC	Total Body Composition
WR	Well Recovered
