# Supplementary material for: Assessment of Bone Mineral Density, Total Body Composition and Joint Integrity in Long COVID: A 12-Month Longitudinal Feasibility Study
Source: J Clin Med. 2025 Dec 2;14(23):8558. doi: 10.3390/jcm14238558 (PMC12692885; doi:10.3390/jcm14238558)
Supplement: Supplementary file 1 [file jcm-14-08558-s001.zip › jcm-3997229-supplementary.pdf]

Supplementary

Table S.1:

Intra-Rater Reliability of Ultrasound Scoring Across Joints			
	**Hypertrophy**	**Effusion**	**Power Doppler**
**Metacarpal Interphalangeal Joints**	0.66	0.77	0.74
**Proximal Interphalangeal Joints**	0.88	0.1	0.88
***Knee Joint**	0.66	0.1	0.1

*S 1: Intra-Rater Reliability of Ultrasound Scoring Across Joints; *Unweighted kappa statistics*

Table S.2:

Baseline and Follow-up BMD (g/cm²) Measured by DXA in LC and WR Participants, Stratified by Sex.

Region	Side	***Baseline*** Sex, (*n*) (WR/LC)	WR	LC	*p*	***Follow-up*** Sex, (*n*) (WR/LC)	WR	LC	*p*
**Total body**	-	Female (18/38)	1.148±0.09	1.208±0.097	0.032	Female (15/29)	1.139±0.104	1.203±0.104	0.061
		Male (21/7)	1.279±0.125	1.302±0.075	0.658	Male (15/7)	1.291±0.139	1.285±0.077	0.914
**L1-L4**	-	Female (14/27)	1.146±0.034	1.200±0.028	0.248	Female (10/23)	1.127±0.144	1.184±0.157	0.336
		Male (18/6)	1.298±0.257	1.224±0.06	0.509	Male (13/5)	1.315±0.300	1.250±0.119	0.651
**Femoral neck**	Rt	Female (18/38)	0.919±0.126	0.964±0.129	0.234	Female (15/29)	0.904±0.117	0.967±0.146	0.153
		Male (21/7)	1.009±0.148	1.033±0.155	0.707	Male (15/7)	1.018±0.15	1.031±0.124	0.853
	Lt	Female (18/38)	0.912±0.142	0.974±0.157	0.155	Female (15/29)	0.9±0.139	1.003±0.243	0.134
		Male (21/7)	1.019±0.15	0.985±0.130	0.595	Male (15/7)	1.031±0.151	0.969±0.115	0.349
**Total hip**	Rt	Female (18/38)	0.959±0.145	1.012±0.123	0.164	Female (15/29)	0.941±0.136	1.015±0.142	0.104
		Male (21/7)	1.082±0.154	1.089±0.201	0.933	Male (15/7)	1.105±0.174	1.087±0.189	0.827
	Lt	Female (18/37)	0.945±0.15	1.010±0.129	0.099	Female (15/27)	0.928±0.141	1.010±0.148	0.086
		Male (21/7)	1.09±0.167	1.055±0.171	0.642	Male (15/7)	1.115±0.185	1.048±0.159	0.419

*S 2: Baseline and Follow-up BMD (g/cm²) Measured by DXA in LC and WR Participants, Stratified by Gender. BMD: bone mineral density (g/cm2); L1-L4: lumbar spine; Rt: Right; Lt: Left; WR: Well-recovered; LC: long COVID; (n): participants number completed DXA scan at each timepoints; p-values based on paired t-test; BMD values reported as mean ± SD * Statistically significant at p<0.01.*

Table S.3:

Sex Stratified Within LC and WR Group BMD Changes (g/cm²) from Baseline to Follow-up.									
**Region**	**Side**	**Sex, (*n*)**	**WR**			**Sex, (*n*)**	**LC**		
			Baseline	***Follow-up***	***p***		***Baseline***	***Follow-up***	***p***
**Total body**	-	Female (n=15)	1.139±0.094	1.139±0.104	0.974	Female (29)	1.205±0.105	1.203±0.104	0.492
		Male (n=15)	1.293±0.143	1.291±0.139	0.774	Male (7)	1.302±0.075	1.285±0.077	0.013
**L1-L4**	-	Female (n=10)	1.1412±0.136	1.127±0.144	0.145	Female (n=19)	1.181±0.145	1.165±0.153	0.101
		Male (n=13)	1.307±0.297	1.315±0.3	0.475	Male (n=5)	1.255±0.14	1.250±0.119	0.904
**Femoral neck**	Rt	Female (n=15)	0.900±0.116	0.904±0.117	0.546	Female (n=29)	0.972±0.141	0.967±0.146	0.485
		Male (n=15)	1.015±0.158	1.018±0.15	0.712	Male (n=7)	1.033±0.155	1.031±0.124	0.834
	Lt	Female (n=15)	0.901±0.137	0.9±0.139	0.860	Female (n=29)	0.974±0.173	1.003±0.243	0.103
		Male (n=15)	1.039±0.155	1.031±0.151	0.198	Male (n=7)	0.985±0.13	0.969±0.115	0.079
**Total hip**	Rt	Female (n=15)	0.938±0.135	0.941±0.136	0.521	Female (n=29)	1.013±0.13	1.015±0.142	0.696
		Male (n=15)	1.093±0.173	1.105±0.174	0.024	Male (n=7)	1.089±0.201	1.087±0.189	0.886
	Lt	Female (n=15)	0.927±0.138	0.928±0.141	0.857	Female (n=27)	1.006±0.141	1.010±0.148	0.226
		Male (n=15)	1.109±0.184	1.115±0.185	0.192	Male (n=7)	1.055±0.171	1.048±0.159	0.288

*S 3: BMD: bone mineral density (g/cm2); L1-L4: lumbar spine; Rt: Right; Lt: Left; WR: Well-recovered; LC: long COVID; (n): participants number completed DXA scan at both timepoints; p-values based on paired t-test; BMD values reported as mean ± standard deviation (SD); * Statistically significant at $p<0.01$.*

Table S.4:

Baseline and Follow-up Total Body Composition by DXA in LC and WR Participants, Stratified by Sex.

Region	Baseline				Follow-up			
	**Sex**	**WR**	**LC**	***p***	**Sex**	**WR**	**LC**	***p***
**Gynoid Region Fat (%)**	Female	0.431±0.080	0.490±0.076	0.016	Female	0.433±0.083	0.493±0.062	0.024
	Male	0.377±0.090	0.372±0.058	0.852	Male	0.364±0.079	0.380±0.070	0.597
**Gynoid Tissue Fat (%)**	Female	0.441±0.081	0.500±0.076	0.015	Female	0.433±0.083	0.493±0.062	0.023
	Male	0.386±0.091	0.380±0.059	0.936	Male	0.365±0.080	0.380±0.070	0.597
**Gynoid Fat Mass (g)**	Female	2103±1027	3272±1756	0.012	Female	2009±781	3247±1674	0.025
	Male	3415±1484	4629±1843	0.094	Male	3452±1679	4669±1765	0.112
**Gynoid Lean Mass (g)**	Female	5964±689	6200±1158	0.686	Female	5774±576	6134±1186	0.527
	Male	8214±1605	8702±1174	0.326	Male	8623±1487	8803±1359	0.698
**Android Region Fat (%)**	Female	0.393±0.115	0.477±0.120	0.006*	Female	0.404±0.104	0.486±0.098	0.014
	Male	0.453±0.080	0.485±0.058	0.300	Male	0.439±0.082	0.490±0.062	0.162
**Android Tissue Fat (%)**	Female	0.393±0.115	0.477±0.120	0.006*	Female	0.404±0.104	0.486±0.098	0.014
	Male	0.453±0.080	0.485±0.058	0.300	Male	0.439±0.082	0.490±0.062	0.162
**Android Region Fat Mass (g)**	Female	2103±1027	3272±1756	0.012	Female	2009±781	3247±1674	0.025
	Male	3415±1484	4629±1843	0.094	Male	3452±1679	4669±1765	0.113
**Legs Tissue Fat (%)**	Female	0.394±0.097	0.460±0.091	0.017	Female	0.396±0.101	0.462±0.079	0.021
	Male	0.336±0.102	0.323±0.075	0.978	Male	0.324±0.100	0.330±0.081	0.895
**Legs Lean Mass (g)**	Female	13380±1687	13548±2588	0.725	Female	12876±1376	13376±2672	0.891
	Male	18536±3175	19834±2792	0.410	Male	18850±2369	19818±2636	0.417
**Total Lean Mass (g)**	Female	41309±5109	42438±7484	0.944	Female	39464±3715	42088±7625	0.594
	Male	55432±8571	61006±8440	0.193	Male	57319±7564	60989±8103	0.378

*S.4: WR: Well-recovered; LC: long COVID; Number of participants number completed DXA scan at both timepoints Female [Baseline (WR=18, LC=38) and Follow-up (WR=15, LC=29)], Male [Baseline (WR=21, LC=7) and Follow-up (WR=15, LC=7)] ; p-values using appropriate tests (Mann–Whitney U or independent t-tests) based on data normality; Data are presented as mean ± standard deviation (SD); * Statistically significant at p<0.01*

Table S.5:

Sex Stratified Within LC and WR Group Changes Body Composition from Baseline to Follow-up.								
**Region**	***Sex, n (15/15)***	***WR***			**Sex, *n* (29/7)**	**LC**		
		Baseline	***Follow-up***	***p***		***Baseline***	***Follow-up***	***p***
**Gynoid Region Fat (%)**	Female	0.431±0.079	0.433±0.083	0.690	Female	.487±.075	0.493±0.062	0.127
	Male	0.372±0.079	0.364±0.079	0.733	Male	.372±.058	0.380±0.070	0.236
**Gynoid Tissue Fat (%)**	Female	0.440±0.081	0.433±0.083	0.211	Female	.497±.075	0.493±0.062	0.230
	Male	0.381±0.079	0.365±0.080	0.139	Male	.380±.059	0.380±0.070	1.000
**Gynoid Fat Mass (g)**	Female	4740±1508	4790±1633	0.233	Female	6440±2425	6487±2270	0.117
	Male	5634±2554	5424±2520	0.909	Male	5512±1769	5807±2074	0.042
**Gynoid Lean Mass (g)**	Female	5795±577	5774±576	0.776	Female	6160±1168	6134±1186	0.689
	Male	8677±1540	8623±1487	0.334	Male	8702±1174	8803±1359	0.865
**Android Region Fat (%)**	Female	0.387±0.110	0.400±0.104	0.030	Female	.474±.116	0.481±0.098	0.176
	Male	0.447±0.074	0.435±0.082	0.460	Male	.482±.058	0.487±0.062	0.310
**Android Region Fat Mass (g)**	Female	1938±807	2009±781	0.026	Female	3241±1699	3247±1674	0.256
	Male	3647±1641	3452±1679	0.363	Male	4629±1843	4669±1765	0.865
**Android Tissue Fat (%)**	Female	0.391±0.111	0.404±0.104	0.030	Female	0.479±0.116	0.486±0.098	0.198
	Male	0.452±0.074	0.439±0.082	0.460	Male	0.485±0.058	0.490±0.062	0.310
**Legs Tissue Fat (%)**	Female	0.392±0.094	0.396±0.101	0.125	Female	0.455±0.090	0.462±0.079	0.028
	Male	0.335±0.097	0.324±0.100	0.495	Male	0.323±0.075	0.330±0.081	0.176
**Legs Lean Mass (g)**	Female	12919±1272	12876±1376	0.909	Female	13528±2645	13376±267	0.393
	Male	19349±3079	18850±2369	0.099	Male	19834±2792	19818±2636	0.865
**Total Lean Mass (g)**	Female	39676±3617	39464±3715	0.280	Female	42366±7809	42088±7625	0.455
	Male	57843±7737	57319±7564	0.211	Male	61006±8440	60989±8103	1.000

S. 5: *Results shown separately for female and male subgroups. WR: Well-recovered; LC: long COVID; (n): participants number completed DXA scan at both timepoints; p-values based on Wilcoxon signed-rank; values reported as mean ± standard deviation (SD) at baseline and follow-up; * Statistically significant at p<0.01.*
